# From movement to motivation: a proposed framework to understand the antidepressant effect of exercise

**DOI:** 10.1038/s41398-024-02922-y

**Published:** 2024-07-04

**Authors:** E. J. Hird, A. Slanina-Davies, G. Lewis, M. Hamer, J. P. Roiser

**Affiliations:** 1 Institute of Cognitive Neuroscience, University College London, London, UK; 2 Division of Psychiatry, University College London, London, UK; 3 Institute of Sport, Exercise and Health, University College London, London, UK

**Keywords:** Learning and memory, Human behaviour, Depression

## Abstract

Depression is the leading cause of disability worldwide, exerting a profound negative impact on quality of life in those who experience it. Depression is associated with disruptions to several closely related neural and cognitive processes, including dopamine transmission, fronto-striatal brain activity and connectivity, reward processing and motivation. Physical activity, especially aerobic exercise, reduces depressive symptoms, but the mechanisms driving its antidepressant effects are poorly understood. Here we propose a novel hypothesis for understanding the antidepressant effects of exercise, centred on motivation, across different levels of explanation. There is robust evidence that aerobic exercise decreases systemic inflammation. Inflammation is known to reduce dopamine transmission, which in turn is strongly implicated in effort-based decision making for reward. Drawing on a broad range of research in humans and animals, we propose that by reducing inflammation and boosting dopamine transmission, with consequent effects on effort-based decision making for reward, exercise initially specifically improves ‘interest-activity’ symptoms of depression—namely anhedonia, fatigue and subjective cognitive impairment - by increasing propensity to exert effort. Extending this framework to the topic of cognitive control, we explain how cognitive impairment in depression may also be conceptualised through an effort-based decision-making framework, which may help to explain the impact of exercise on cognitive impairment. Understanding the mechanisms underlying the antidepressant effects of exercise could inform the development of novel intervention strategies, in particular personalised interventions and boost social prescribing.

## Introduction

Depression is the leading cause of disability worldwide [1]. In addition to persistent low mood, core features of depression include anhedonia, fatigue and subjective cognitive impairment, known collectively as ‘interest-activity’ symptoms [2]. These symptoms can prevent people from engaging in work, disrupt social relationships and curtail the pleasure people take in life. Depression is also difficult to treat: fewer than half of people respond to selective serotonin reuptake inhibitors, with only around one-third remitting from their first pharmacological treatment [3] and still fewer remit after switching to a second treatment [4]. Even individuals with apparently similar symptoms of depression may respond differently to the same treatment, probably because depression is highly mechanistically heterogeneous [5].

Impaired motivation is an important feature of depression that is associated with interest-activity symptoms [6] and linked to poor treatment outcome [6]. Specifically, patients with depression - particularly those with anhedonia - have impaired reward-processing [7] and are less willing to exert effort in order to obtain reward [8–10]. Given that dopamine function is associated with anhedonia [11, 12], and with the propensity to exert effort in both animals [13] and humans [14] (with greater effects of dopamine manipulation in individuals with lower dopamine transmission [15]), a candidate alternative to SSRIs to treat depression is to use medications that enhance dopamine transmission. However, with the exception of bupropion [16], trials using dopaminergic agents have had limited success, likely due to the mechanistic heterogeneity of depression which may render a one-size-fits-all treatment approach ineffective [17].

Several studies indicate that dopamine transmission is enhanced by physical activity, in particular aerobic exercise [18, 19], indicating this as a potentially useful alternative method to boost dopamine and increase motivation in depression. Indeed, it is well established that any level of physical activity can help prevent [20] and treat [21, 22] depression in both adults and adolescents, with a longer duration of intervention and more intense activity having a greater effect [23]. Objectively measured cognitive impairment in depression, which is not improved by most serotonergic antidepressants [24], has also been reported to be improved by physical activity [25, 26], although the picture is more mixed than for subjective depressive symptoms such as mood. Cognitive impairment in depression is especially important and under-studied, representing a clear unmet clinical need, as it can have a profound impact on functioning and many depressed individuals struggle with enduring cognitive impairment even after other symptoms resolve [27].

Reduced inflammation is likely to be just one of several mechanisms through which exercise has an antidepressant effect. Substantial prior research indicates that exercise alters several biological processes that could influence depressive symptoms [28], such as elevated brain derived neurotrophic factor (BDNF) [29], increased grey matter volume [30], decreased oxidative stress [31] and altered neuroendocrine responses [32, 33]. Understanding the mechanisms driving the effect of physical activity on symptoms of depression could allow exercise programmes to be made more accessible, effective and individually tailored to patients likely to respond to physical activity as an intervention. However, although there has been extensive research on the biological, neural and behavioural effects of exercise in animal models of depression, to date the mechanisms driving its antidepressant effects in humans are poorly understood [34, 35].

In this review we outline a novel framework for understanding the antidepressant effect of physical activity, especially aerobic exercise, in depression. We initially summarise possible mechanisms underlying the motivational symptoms of depression - including dopamine-driven changes in effort-based decision making and associated brain circuitry - and their relationship with inflammation. We then propose a pathway whereby through reducing inflammation and consequently boosting dopamine transmission, with downstream effects on brain circuitry involved in decision making and reward processing, exercise primarily alters interest-activity symptoms of depression by increasing propensity to exert effort to obtain reward. We hypothesise that exercise also treats cognitive impairment in depression through altering effort-related brain circuitry, thereby increasing propensity to exert cognitive as well as physical effort.

## Motivational dysfunction in depression

### Motivation, reward processing and effort-based decision making

Apathy and anhedonia are two important motivational symptoms. Apathy refers to a loss of enthusiasm for a variety of activities, which is often expressed as lower motivation to perform goal-directed behaviour across different domains of function [36]. Apathy is commonly seen in mental health conditions such as depression and schizophrenia and neuropsychiatric conditions such as Alzheimer’s and Parkinson’s disease [2]. Anhedonia is defined as diminished interest or pleasure in previously enjoyable activities and is a core part of the interest-activity cluster of depressive symptoms [37]. It has been hypothesised that both apathy and anhedonia are driven by an impairment in reward processing [38–42]. Depressed individuals with high anhedonia show poorer reward learning compared to those with low anhedonia [43, 44], as well as lower reward sensitivity [45], even following remission [46]. However, one difficulty in interpreting his literature is that many tasks used to measure reward learning do not dissociate reward learning from reward value (the internal expectation assigned to potentially rewarding outcomes [47]).

The use of computational modelling to decompose different parameters contributing to behaviour is helpful to distinguish between parameters of reward processing; for example, one study showed that anhedonia was associated with lower inverse temperature (often interpreted as reflecting lower reward value) rather than lower learning rates [45]. More research applying computational models to reward learning paradigms is required to dissociate these features of reward processing. More broadly, reward processing is known to be disrupted in depression [7], with case-control differences most reliably observed on tests of reward bias (assessed through an asymmetrically rewarded signal detection task), cost-benefit decision making (how potential rewards are used to guide decisions) and reinforcement learning (updating expectations from outcomes to guide future choices) [48]. At the neural level, these changes are thought to be driven by altered activation during reward processing in the ventral striatum, caudate and anterior mid-cingulate cortex (aMCC, often referred to as dorsal anterior cingulate cortex (dACC), or simply ACC) in depression [49–52].

Anhedonia is associated with disruption to both dopamine function [53, 54] and striatal activation [52], consistent with preclinical studies implicating the mesolimbic dopamine system in motivational dysfunction [55, 56]. However, the mechanisms driving anhedonia are not well understood, arguably because it is driven by a variety of cognitive processes that have not been fully characterised and dissociated [57]. One potential mechanism is an increased sensitivity to the expenditure of effort to gain reward. Accordingly, depressed patients are less willing to exert effort for reward, are less able to use information about reward probability and magnitude to guide choices and performance on effort-based decision-making tasks is associated with the duration of depressive episodes [8–10]. Self-reported anhedonia correlates with various markers of reward processing, including behaviour and neural activation during effort-based decision-making [9, 58, 59], the ability to sustain an optimal reward response over time [44, 60], reinforcement learning [43, 61] and reward sensitivity [45]. However, based on the extant literature it is difficult to conclude that this association is truly specific to anhedonia as opposed to other symptoms of depression, as only a minority of studies have reported associations between anhedonia and reward processing that survive controlling for overall symptom severity; although this has been shown in some cases, for example reward prediction error in the striatum [62], reward learning [61] and reward response over time [44]. Anhedonia is especially important to understand because it is associated with antidepressant treatment failure [6], a more chronic course of depression [63, 64] and fewer depression-free days following antidepressant treatment [65]. Antidepressants are generally ineffective for treating motivational dysfunction and can even exacerbate these symptoms in some patients, indicating an important unmet clinical need [66–68].

A common method used to assess effort-based decision-making is to vary the level of physical effort required to obtain reward, as in the Effort-Expenditure for Rewards Task (EEfRT) [69]. In this task, physical effort is manipulated by the number of button presses required in a fixed amount of time and the potential reward that can be obtained is varied [69]. Motivation is indexed by the proportion of trials on which participants opt for the ‘high effort/high reward’ option, relative to the ‘low effort/low reward’ option, similar to analogous tests in rodents [70]. Studies using the EEfRT have revealed lower willingness to expend effort for reward in patients with subsyndromal depression, first-episode depression and recurrent major depressive disorder, compared to controls, particularly in those with high anhedonia [8, 9]. Importantly, choices to engage on the EEfRT also correlate with trait anhedonia in healthy participants [69]. Another physical effort task is the grip-force task: participants are required to squeeze a gripper at different intensities to win differing levels of reward. Healthy participants with high apathy traits (a motivational impairment which overlaps with anhedonia [42]) show an increased modulation of grip force by effort level [71].

However, the above results are confounded by physical ability (either button-pressing speed or strength). One way to overcome this confound is to calibrate the required level of physical exertion to each individual participant and examine the decision to accept or reject a challenge, instead of comparing high vs low effort options. In such designs participants decide on each trial whether to accept an effortful challenge to win varying amounts of reward, as in the Apple Gathering Task (AGT) [72]. Motivation on this task is increased when Parkinson’s disease (PD) patients take their usual dopaminergic medication, highlighting the strong link between dopamine transmission and effort-based decision making [73]. Further, healthy participants with high apathy scores are more sensitive to effort (less likely to accept effort trials) on the AGT [74].

### Cognitive control

Cognitive control (often called executive function) describes a set of effortful cognitive processes [75] which allow for flexible adaptation of behaviour in line with goals [76], including functions such as working memory, task-switching, response inhibition and attentional control [77]. Cognitive control is impaired in depression [77, 78] which may be in part due to changes in motivation - cognitive control is typically experienced as effortful [75] which could explain why people with depression avoid exerting cognitive control. Poorer performance on tests of cognitive control in depression is accompanied by other cognitive impairments, including on selective attention and memory tasks, which are not resolved by standard antidepressant treatment [79]. One possible explanation for such a broad pattern of impairment is that reduced cognitive control, arising from a failure to allocate cognitive effort, causes more general impairments in cognitive processing [79–82]. Reduced cognitive control has also been proposed as a potential mechanism driving other symptoms of depression, including indecisiveness, negative automatic thoughts, poor concentration and distorted cognitive processing [83].

### Neural correlates of effort-based decision making

**Fig. 1:**
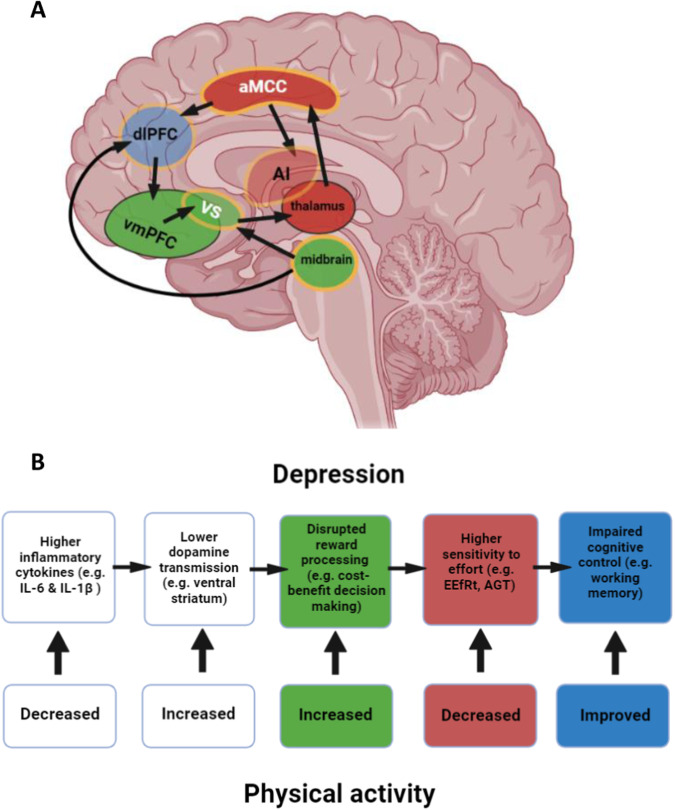
A mechanistic pathway for depression which is modulated by physical activity. **A** Areas signalling reward (green), effort (red) and involved in cognitive control (blue), with areas involved in integrating reward and effort outlined in yellow. White text indicates known modulation by physical activity, especially aerobic exercise. Not all anatomical connections are depicted for reasons of clarity. Light shading (AI, VS, dlPFC) indicates that the region is situated more laterally than the slice depicted. **B** Cognitive processes corresponding to the neural changes illustrated in (**A**), and how different components implicated in effort-based decision making for reward are affected by exercise. aMCC anterior mid-cingulate cortex, dlPFC dorsolateral prefrontal cortex, vmPFC ventromedial prefrontal cortex, VS ventral striatum, AI anterior insula, IL-6 interleukin 6, IL-1β interleukin-1 beta, EEfRT effort-expenditure for rewards task, AGT apple gathering task.

In humans, lesions in the basal ganglia [84, 85] and dmPFC [86, 87] are associated with a motivational deficits such as inertia and apathy, indicating the importance of these structures in signalling motivation. Motivated behaviour is signalled by an interconnected network of regions with the aMCC and ventral striatum playing key roles, demonstrated by the importance of these structures in apathy across clinical disorders [88] (Fig. 1A). A meta-analysis of functional magnetic resonance imaging (fMRI) studies showed that the aMCC and anterior insula (AI) are central to signalling effort [89]. As well as the aMCC and AI, activity in the striatum, ventral tegmental area and posterior parietal cortex have been found to signal actual and expected effort [90–93]. Direct electrical stimulation of the aMCC (during brain surgery) induces a feeling of imminent challenge [94] and in line with the importance of the aMCC in motivated behaviour, apathy is associated with increased effort sensitivity and with decreased connectivity between this region and the supplementary motor area [74].

Meta-analysis of fMRI studies has shown that whilst the aMCC and AI signal effort, the vmPFC, ventral striatum and midbrain chiefly signal reward value [89] (Fig. 1A). Specific parts of these circuits appear to integrate effort and reward to compute a net value of performing an action [48, 89]; effort and reward expectations are integrated in the ventral striatum, midbrain and aMCC [90, 95, 96]. Reward devaluation by both cognitive and physical demands is signalled in the aMCC and AI, as well as the dorsolateral prefrontal cortex (dlPFC) and intraparietal sulcus [97]. The supplementary motor area and aMCC have also been shown to encode the difference in reward and effort between chosen and unchosen options, providing a signal to guide decisions about whether to engage in an effortful challenge [98].

The expected value of control (EVC) theory proposes that cognitive control is modulated by a decision-making process weighing up the potential gain of control against its cost [99]. EVC is calculated based on its efficacy (probability of an outcome, similar to confidence), value, and effort cost. The aMCC is thought to compute this decision-making process, and to signal to other regions such as the dlPFC to implement control (Fig. 1A); accordingly dlPFC activity is reduced in depression [100–102], including during cognitive control [103] and activity in the dlPFC predicts improvements in depressive symptoms after non-invasive brain stimulation treatment [104]. In line with an overlap between effort-related and control-related processes, the neural circuits implicated in the allocation of effort [105] and cognitive control [104] converge on a circuit known as the ‘cingulo-opercular network’ (CON, often termed the ‘salience network’ in the resting-state fMRI literature) which includes the aMCC, AI, ventrolateral thalamus and medial caudate [106–108], indicating shared functionality between effort allocation and cognitive control.

### Dopamine, anhedonia and effort-based decision-making

Dopamine is associated with anhedonia, consistent with the link between anhedonia and motivation; indeed, it is well established that dopamine plays a central role in motivation [11, 48, 56, 109]. Molecular imaging studies indicate that D2/3 receptor availability in the ventral striatum correlates negatively with anhedonia in depressed patients [12] and with lower dopamine transporter binding in the striatum, caudate and putamen in depressed patients with anhedonia compared to controls [110]. In patients with Parkinson’s disease, anhedonia and apathy are negatively correlated with striatal dopamine transporter binding, especially as neurodegeneration progresses [11].

There is substantial evidence that dopamine transmission influences effort exertion. Administration of amphetamine, which directly releases dopamine, increases willingness to exert effort [111], although interestingly one animal study showed that while lower doses of amphetamine increased high-effort choices, higher doses had the inverse effect, decreasing high-effort choices [112]; indicating that the relationship between dopamine and effort is complex, possibly corresponding to the well-documented ‘inverted U-shaped’ function of dopaminergic modulation of PFC functioning [113]. In animals, dopamine D2 receptor antagonism decreases effort expenditure [112, 114] specifically in the nucleus accumbens (equivalent to the ventral striatum in humans) [70]. In healthy humans, individual differences in dopamine release in the striatum and vmPFC, measured with positron emission tomography (PET), were associated with willingness to expend effort [14]. This result is consistent with animal research showing that dopamine activity in the nucleus accumbens is greater in high-effort responders [115]. Effort is typically considered to be signalled by tonic dopamine transmission [56, 116], but when studied across multiple timescales in rodents, tonic (minute-by-minute) dopamine release correlates with both reward rate and motivational vigour, whilst phasic (second-by-second) dopamine release encodes expected reward, suggesting that dopamine may encode a single decision variable representing the available reward for investment of effort across multiple timescales [109].

### Inflammation, anhedonia, dopamine and effort-based decision-making

There is good evidence that the motivational symptoms of depression are related to inflammation. Systemic inflammation is associated with increased odds of developing depression [117] and is dysregulated in depression [118], particularly in those with pronounced anhedonia [119]. Conversely, inhibition of inflammatory cytokines reduces depressive symptoms in patients with inflammatory conditions [120] and reduces anhedonia in depressed individuals with high inflammatory cytokines as well as in those with inflammatory conditions [121, 122]. Further, chronic inflammation is thought to result in cognitive impairment and is associated with poorer cognitive control in depression [123–125]. It is worth noting that findings into the antidepressant effects of anti-inflammatory medication are mixed [126], and although a meta-analysis found an overall antidepressant effect [127], important methodological limitations remain: in particular, because increased inflammation is only present in a subset of depressed individuals [128], participants should ideally be stratified by their inflammatory status at baseline and inflammatory markers should be assessed pre-and post-treatment. Further, given evidence for a link between anhedonia and inflammation [119], it is important to assess changes in anhedonia specifically, alongside depressive symptoms [129].

Substantial research suggests that dopamine activity is affected by peripheral inflammation. A recent study showed that depressed participants with high levels of inflammation who received the dopamine precursor levodopa had increased functional connectivity between the ventral striatum and vmPFC. In this study a decrease in anhedonia after levodopa was correlated with ventral striatum-vmPFC connectivity only in patients with high levels of inflammation [130], underscoring the link between inflammation, dopamine functioning, brain circuitry and anhedonia. Administration of levodopa has also been shown to reverse the effect of inflammation on striatal dopamine release in non-human primates [131]. Dysregulation of inflammation in depression has knock-on effects on reward processing: transiently increasing inflammatory cytokines such as interleukin-6 (IL-6) and tumour necrosis factor-alpha (TNF-α) decreases reward responsivity in the ventral striatum, as well as dopamine transmission in the caudate and putamen and increases depressive symptoms including anhedonia [132, 133]; and ventral striatal response to reward anticipation has been reported to mediate the effect of inflammation on mood [134].

In line with a link between inflammation and effort sensitivity, several animal studies have indicated that inducing inflammatory responses increases effort sensitivity and depression-like behaviours [135–137]. In humans, induced inflammation resulted in a greater sensitivity to the probability of winning when choosing to exert effort, which was also related to sleepiness [138] and in another study, inflammation increased effort sensitivity [139]. A recent study reported that depressed participants with high levels of inflammation who received levodopa did not show a decrease in effort sensitivity assessed by behaviour on the EEfRT [130], but these results may have been affected by the completion of the EEfRT after the peak concentration of levodopa had passed; additionally, this study did not include a healthy control group comparison, limiting interpretability.

### How does aerobic exercise treat motivational symptoms of depression?

There are various mechanisms by which exercise might treat symptoms of depression, which are not mutually exclusive [28]. Exercise has been shown to increase BDNF [29] which may mediate the effect of exercise on mood and cognition, albeit this effect is stronger on acute measures of BDNF immediately after exercise (ES ~ 0.5) than on resting BDNF after regular programmed exercise (ES ~ 0.3) [29]. Further, the effect of exercise on BDNF has been examined mainly in animals due to the difficulty of measuring cellular and molecular changes in human brains [140]. There is also preliminary evidence that exercise increases cortical volume [30] and decreases oxidative stress [31]. There is mixed evidence for a modulation of the neuroendocrine system in depressed individuals by exercise [33], including decreased adrenocorticotrophic hormone [32] and cortisol levels [33] which can be dysregulated in depression [141]. Exercise also has psychological benefits; it enhances self-esteem [142] and self-efficacy [143–145] which could mediate its antidepressant effects [146], underlining the importance of assessing the effects of exercise at multiple levels of explanation.

We have summarised a possible pathway driving interest-activity symptoms of depression, whereby inflammation reduces dopamine transmission, increasing effort sensitivity and thereby anhedonia and fatigue, which can also explain cognitive impairment. In this next section, we present evidence that physical activity, especially aerobic exercise, modulates each stage in this process and propose that this represents one pathway by which such interventions reduce depressive symptoms (Fig. 1).

### Antidepressant effects of physical activity

There is good meta-analytic evidence from observational studies indicating that individuals with higher levels of physical activity have lower odds of developing depression [20]; for example, those exercising for at least four hours per week had 18% lower risk of depression, with a 25% reduction in those who exercised for at least nine hours per week [147]. Based on such evidence it has been estimated that 12` of incident depression could be prevented if all adults exercised for at least nine hours per week [147], albeit this figure is derived from observational studies, which may be confounded and therefore needs to be interpreted with caution. Another meta-analysis suggested that increasing levels of moderate-to-vigorous physical activity is negatively associated with the incidence of depression as well as the occurrence of subclinical symptoms [148].

Consistent with a causal effect, meta-analyses of randomised controlled trials of aerobic exercise interventions estimate a substantial effect on overall depressive symptoms [149, 150], in both adults (ES ~ 0.8) [21] and young people (ES ~ 0.8) [22], although a lower effect size was reported in a meta-analysis including less vigorous interventions (ES ~ 0.3) [151]. Another meta-analysis found a moderate effect in favour of exercise combined with standard treatments (namely medication and psychotherapy), with the greatest benefits observed in more severely depressed patients [152]. It is important to note that it is more difficult to blind participants in exercise studies than in drug studies because of the physical nature of the intervention; however, it has been reported that participants have similar efficacy expectations for aerobic versus non-aerobic activities (such as stretching or toning exercises) [153], making any differences between an aerobic active group and a non-aerobic stretching control group less likely to be due to the placebo effect. Additionally, the standardised effect size of the antidepressant effect of exercise in the most rigorously controlled trial (ES ~ 0.7) [21] is numerically higher than for the most common psychological therapies (ES ~ 0.5) [154], which if anything face even greater challenges with blinding. Withdrawing regular physical activity increases negative mood in people who do not have depression [155], which appears to be related to changes in an inflammatory marker, IL-6 [156]. A recent review summarised the meta-analyses and systematic reviews assessing the antidepressant effects of exercise [157], concluding that it has a moderate-to-large antidepressant effect when compared to no-treatment or control groups [152, 158–160], is no less effective than antidepressant medication or psychological therapy [158, 159] and has a moderate benefit over treatment as usual [159]. One challenge with using exercise as a treatment for depression may be that for many patients, their symptoms can make it more difficult to engage with exercise, due to low motivation [161–163], fatigue [161, 162], or low mood [161, 162, 164, 165]. Additional barriers to exercise in depressed individuals include a lack of support [164], low confidence in ability [161] and fear of injury [161].

### Physical activity reduces inflammation

Inflammation is a complex process involving multiple biological pathways across different tissues and systems. The physiological responses to exercise are similarly complex and vary depending on the type, frequency and intensity of the exercise performed. There are also differences between the acute response to a single bout of physical activity and the long-term adaptations which occur in response to a training programme (repeated bouts of aerobic exercise over a prolonged period).

Despite this complexity, it is widely accepted that physical activity has a net anti-inflammatory effect, as evidenced by a range of biomarkers [166]. There are multiple mechanisms by which physical activity may reduce inflammation. Whilst inflammation may be mediated by physiological adaptations such as regulating autonomic function and reducing allostatic load, it is also directly related to muscle contraction [167]. Muscle is increasingly recognised as an endocrine organ because it secretes cytokines (or myokines) to coordinate immediate responses to exercise, indicating the importance of the musculature in the anti-inflammatory effects of exercise [167–169]. The anti-inflammatory effects of exercise are also mediated via changes to adipose tissue, as adipocytes can be pro-inflammatory [170]. It is not the overall volume of adiposity per se which has an inflammatory effect, but rather the size of individual adipose cells and their location, with hypertrophic cells and visceral location having the biggest effect [171, 172]. Intervention studies indicate that exercise exerts an anti-inflammatory effect over adipose tissue, promoting smaller adipocyte size and loss of visceral fat [173]. Importantly, this effect is seen over and above weight loss [173].

Whilst there is robust evidence from observational studies of a negative relationship between physical activity and inflammation, results from intervention studies are more mixed, although the overall pattern is that exercise interventions tend to yield an anti-inflammatory effect [174]. An anti-inflammatory effect is observed most consistently in men, which could be due to differences in adiposity between the sexes [175], or cyclical variations in baseline levels of inflammation in women which may confound results [176]. It is also important to note that the menstrual cycle and associated fluctuations in hormones may interact with other variables that might affect the antidepressant effect of exercise, such as dopamine function [177] and exercise performance itself [178], though limited human studies and heterogeneous study designs make it difficult to draw firm conclusions. While exercise has an anti-inflammatory effect, a sedentary lifestyle is independently associated with chronic low-grade inflammatory illnesses [179, 180]. It is thus important to assess an individual’s daily activity levels in addition to their engagement in structured exercise when evaluating the effects of any intervention [179, 180].

Further complications in exercise intervention studies relate to the heterogeneity in inflammatory markers and the physical activity intervention under examination. Both the acute and chronic responses to aerobic vs resistance training differ, as do the responses to moderate vs high intensity aerobic training [181]. This is not only important when considering which intervention may be most beneficial, but also whether certain exercise regimes could be potentially harmful. Given that participants with depression may already have elevated inflammation [117], the acute stress placed on the body by certain forms of activity (for example high intensity interval training) could potentially be deleterious, negating any potential long term benefits [182]. This underlines the importance of tailoring exercise interventions to the individual. Indeed, while there is increasing evidence for the anti-inflammatory effect of exercise across inflammatory disorders affecting the skeletal system, the cardiovascular system and the nervous system [181, 183], further work is needed to assess which type of exercise intervention—and which intensity, duration and frequency - has the greatest anti-inflammatory effect in depression.

### Physical activity boosts dopamine transmission and neural reward circuitry

Given the evidence that exercise reduces inflammation [184], and the inverse association between inflammation and dopamine function [130], it is plausible that exercise might enhance dopamine transmission [185]. Indeed, animal studies show that exercise protects against the loss of dopamine neurons associated with inflammation [186], elicits dopamine release [187] and increases striatal activity [187]. In one study in Parkinson’s disease patients, a 30-minute session of aerobic exercise resulted in increased striatal dopamine release, more so in habitual exercisers, who also showed an increase in striatal activation to reward as well as lower apathy [18]. The causal effect of physical activity on dopamine functioning has been further explored in a 36-session intervention study, in which Parkinson’s disease patients in the active group showed increased striatal activation to reward and enhanced dopamine release in the caudate nucleus [19], relative to the control group. This is consistent with the hypothesis that exercise alters striatal response to reward, potentially via enhancing transmission in the mesolimbic dopaminergic pathway [19].

Counter-intuitively, some (but not all [188]) neuroimaging studies suggest that exercise results in an acute *decreased* striatal response to reward (i.e. in the immediate aftermath of activity), which might reflect an increase in tonic extracellular dopamine, inhibiting the magnitude of phasic dopamine release [189, 190]. A recent study also examined the effect of eight weeks of aerobic exercise on a reward-related electroencephalography (EEG) component termed the ‘reward positivity’, thought to correspond to ventral striatum-aMCC signalling. Reward positivity was not changed by the intervention, although its amplitude did predict who experienced an improvement in symptoms: individuals with larger pre-treatment reward positivity were more likely to respond to aerobic exercise, again implicating reward processing in the effect of exercise on depression [191]. The causal effect of exercise on tonic and phasic dopamine function and reward processing over time, and its association with interest-activity symptoms, merit further investigation.

### Physical activity increases effort exertion and decreases anhedonia

Given its impact on both dopamine transmission and reward processing, it seems likely that exercise increases propensity to engage in effort, although few studies have directly tested this hypothesis. An exploratory analysis of one study showed that immediately after a single 20-minute running session, healthy individuals who had been running for more years exhibited increased willingness to exert effort, with the opposite pattern in those who had been running for fewer years [192], suggesting that fitness level interacts with the acute effect of physical exercise on effort sensitivity. The only study that has assessed neural activation during effort-based decision-making after a course of exercise (in this case, a three-month intervention, again in healthy participants) reported reduced effort sensitivity and effort-related aMCC activation; albeit there was no control group which limits interpretability [193]. Further investigation of the effects of exercise on effort-based decision-making, in randomised studies using appropriate control groups, is warranted.

In line with its effects on both effort sensitivity and depression, physical activity is negatively associated with anhedonia [194] and fatigue [195]. A recent study reported that anhedonia was reduced in people with depressive symptoms after an eight-week exercise intervention, although an improvement in general depressive symptoms was also observed, making it difficult to assess the specificity of this finding [196]. A causal relationship between physical activity and motivation was indicated in a 12-week martial arts intervention in children, which yielded large improvements specifically on observer ratings of motivation, perseverance, will and engagement [197, 198]. The beneficial effect of exercise on anhedonia has been further demonstrated in a three-month intervention study as an augmentation treatment for patients with treatment-resistant depression, finding improved anhedonia and other motivational symptoms [199]. Both anhedonia and motivational changes were correlated with change in depression severity, and changes in motivation preceded improvement in overall symptoms, further indicating a directional effect of exercise on anhedonia and mood [199] (Fig. 1B).

### Physical activity improves cognitive control

According to EVC theory, cognitive control results from a decision-making process in which the potential rewards and costs of cognitive control are weighed up and the brain allocates resources accordingly [75, 77]. The CON [89, 106, 108] is thought to play a key role in this process, activated both when deciding to perform an effortful action [105] and during cognitive control tasks [104, 200]; importantly, CON connectivity is also disrupted in depression [201]. This lends credence to the notion that the CON has a central role in the motivational and cognitive symptoms of depression. As discussed above, exercise might reduce CON activity during effort-based decision-making (especially for higher levels of effort) and consequently reduce how challenging a given level of cognitive effort is perceived to be, driving an increased tendency to allocate resources to cognitive control tasks [105].

Consistent with this hypothesis, acute exercise alters PFC function and this varies depending on the level of depressive symptoms [202]. One study showed that exercise indeed reduced aMCC activation and increased dlPFC activation during cognitive control, and improved performance on a flanker task, following a six-month intervention in older adults [203]. However, interventions of similar durations in children yielded only marginal improvements in performance on flanker and antisaccade tasks [200, 204], and *increased* aMCC activity [204] and *decreased* dlPFC activity [200], raising the possibility of age-related differences in the impact of exercise. Indeed, age has been shown to moderate the association between fitness and cognitive performance, with the clearest positive relationships beyond young adulthood [205]. Interestingly, cross-sectional and longitudinal studies suggest that striatal and frontal dopamine transporters and D1 and D2 receptors reduce with age [206–208], which could be one reason that older adults are more sensitive to the psychological effects of exercise.

Behavioural studies also suggest that exercise improves cognitive control [35], though results are less consistent in depression [209]. A meta-analysis of the effect of exercise interventions in healthy older adults revealed robust effects, with moderate-to-large effects on cognitive control tasks (ES = 0.48) [25]. In depression, a recent meta-analysis found that exercise interventions had a beneficial effect on working memory, but nonsignificant effects on inhibition and cognitive flexibility and concluded that there are not yet enough high-quality studies to assess this relationship [209]. Given that it increases cognitive control, exercise might also improve performance on other cognitive processes. While two recent reviews reported that most studies indicate an improvement in cognitive outcomes more broadly after long-term exercise interventions, including in depression [35, 210], many studies in depression have used shorter exercise interventions. In one study, a four-week exercise intervention improved cognitive performance in depressed participants [211], although this was not replicated in another study using a three-week intervention [212]. Other methodological issues in this literature include low levels of attendance at exercise programmes, a lack of blinding and small sample sizes. Further, using wait-list controls could introduce bias due to uncontrolled variables in the control group such as habitual exercise [209].

In summary, preliminary results indicate a potentially beneficial influence of exercise on cognitive control in depression, although studies using randomised designs, appropriate control groups and suitably long intervention durations are required to confirm this. Whether such effects are related to motivational changes, as we hypothesise here, remains unknown. To test this, ideally both cognitive control and effort processing would be assessed throughout an intervention, allowing for time-lagged analyses which could help assess possible mediation of cognitive enhancement by motivational factors.

### Practical applications of the physical activity—inflammation—dopamine—effort framework

Understanding the potential mechanisms underlying the antidepressant effects of exercise in depression—reducing inflammation, enhancing dopamine, and reducing effort sensitivity, consequently improving both interest-activity symptoms and objective cognitive impairment - could inform both nosology and the development of novel intervention strategies. It may provide initial indications of whether there are sub-groups of depressed individuals who are particularly likely to benefit from exercise (for example, individuals with chronic inflammation). This information could also lead to strategies to personalise activity prescription based on motivational factors; for example, psychological interventions such as behavioural activation therapy for individuals with pronounced motivational dysfunction who may struggle to exercise.

Further, individuals are likely to vary in the form of exercise that they most enjoy, and the types of skills they enjoy mastering, which is important to tailor to maximise the likelihood of long-term adherence. This could also pave the way for augmentative approaches, for example combining exercise with psychological interventions. Such knowledge could contribute to social prescribing*—*the use of non-medical interventions to tackle wider influences on health and support individuals to effectively manage their health [213]*—*increasingly a priority for mental healthcare. Information about mechanisms may also make primary care providers more likely to prescribe exercise and encourage policy to establish services.

Exercise is highly scalable, low cost, well suited to early intervention and has beneficial impacts on physical health co-morbidities. Explaining the mechanisms underlying the beneficial effect of exercise on mental health, and the links between them, may help persuade people that exercise is worthwhile for them. This could be important more broadly in addressing the alarmingly low levels of physical activity within the general population. Finally, in the longer term, our ability to develop new interventions for depression will benefit from understanding the mechanisms by which exercise improves symptoms, through back-translation.

## Conclusion

We have summarised a wide range of research indicating that depression, especially anhedonia, is associated with raised levels of inflammatory cytokines and other markers of inflammation, disrupted dopamine transmission and reward processing and in particular lower propensity to exert both physical and cognitive effort. We propose that this may be an important pathway through which exercise exerts an antidepressant effect. The antidepressant effect of aerobic exercise has been convincingly demonstrated through randomised controlled trials, but its mechanism is not well-understood, in part because it likely involves a variety of biological and psychological mechanisms; alongside its effect on inflammation, dopamine and reward processing, exercise also increases neuroplasticity, reduces oxidative stress, alters neuroendocrine levels and improves self-esteem and self-efficacy. Substantial mechanistic research, mainly in animals but with some convergent findings in humans, demonstrates that exercise decreases systemic inflammation which boosts dopamine transmission and increases propensity to exert effort. Future research should continue to utilise randomised controlled trial designs for a rigorous assessment of the antidepressant effects of exercise, in larger samples than tested to date, whilst additionally measuring the effect of exercise on putative mechanistic variables*—*such as inflammation, dopamine transmission and reward processing*—*ideally both at baseline, follow-up and over the course of the intervention, to help assess causality. It would also be important to investigate the potential barriers to implementing exercise, particularly in depressed populations and strategies to encourage exercise use [214]. Understanding the mechanisms underlying the antidepressant effects of physical activity in depression could inform understanding of the mechanisms causing depression as well as the development of novel intervention strategies, in particular personalised intervention and social prescribing.
